# Hypoxia and collagen deposition in the kidneys infected with *Acanthamoeba* sp.

**DOI:** 10.1038/s41598-024-79848-4

**Published:** 2024-11-15

**Authors:** Karolina Kot, Marta Grabowska, Maciej Tarnowski, Patrycja Kupnicka, Patrycja Tomasiak, Danuta Kosik-Bogacka, Natalia Łanocha-Arendarczyk

**Affiliations:** 1 Department of Biology, Parasitology, and Pharmaceutical Botany, Pomeranian Medical University in Szczecin, Szczecin, Poland; 2 Department of Histology and Developmental Biology, Faculty of Health Sciences, Pomeranian Medical University in Szczecin, Szczecin, Poland; 3 Department of Physiology in Health Sciences, Faculty of Health Sciences, Pomeranian Medical University in Szczecin, Szczecin, Poland; 4 Department of Biochemistry, Pomeranian Medical University in Szczecin, Szczecin, Poland; 5 Institute of Physical Culture Sciences, University of Szczecin, Szczecin, Poland

**Keywords:** *Acanthamoeba* sp., Angiogenesis, Collagen, HIFs, Kidneys, TGF-β, Parasitology, Parasite host response

## Abstract

*Acanthamoeba* spp. are facultative, opportunistic pathogens that are found in diverse environments. In the hosts, they lead to multi-organ disease. Recent studies reported that they may induce changes in the kidneys of hosts. The aim of the study was to determine the influence of *Acanthamoeba* sp. on hypoxia and collagen deposition in the kidneys of immunocompetent and immunosuppressed mice infected with *Acanthamoeba* sp. The results strongly suggest that *Acanthamoeba* sp. induces hypoxia in mice with normal and reduced immune response by increasing gene and/or protein expression of HIF1α as well as HIF2α. Additionally, the activation of these factors is probably induced via NOX2/ROS. Hypoxia promotes vessel formation, and we found that angiogenesis occurs in the kidneys of mice infected with the parasite regardless of their immunological status. The proangiogenic factors released in hypoxic conditions cause modulation and inflammation in the kidney cells, which in turn leads to collagen deposition via TGF-β. This work reveals mechanisms occurring in the hosts infected with *Acanthamoeba* sp., highlights as well as supports the relevance of pathophysiology in the kidneys in hosts with systematic acanthamoebiasis.

## Introduction

In eukaryotic cells, the main regulator of oxygen homeostasis and mediator of the hypoxia response is hypoxia-inducible factor 1 (HIF1). It consists of two subunits, α and β, which dimerize to form the HIF complex. HIF1β is a subunit present in the cell regardless of oxygen concentration, whereas HIF1α in normoxic conditions is continuously transferred for degradation by ubiquitination, and during prolonged exposure to hypoxic conditions, it is produced until oxygen homeostasis is restored^1^. HIF1α is expressed in all nucleus-containing cells and contributes to many transcriptional regulatory processes in response to hypoxia. To date, other HIFα isoforms have also been described: HIF2α and HIF3α. HIF2α has a similar structure and function to HIF1α, whereas HIF3α is a negative regulator of HIF1 and it is primarily expressed in lung epithelial cells, contributing to more specialized responses to hypoxia^1,2^. In the kidneys, HIF1α is found in most renal epithelial cells, while HIF2α is mainly expressed in renal interstitial fibroblast-like cells and endothelial cells^3,4^. Both HIF1α and HIF2α are activated by hypoxia but in different phases. HIF1α takes part in the initial adaptation process of hypoxia as it is rapidly induced and then falls to a low level, whereas HIF2α accumulation begins under prolonged hypoxic conditions^3,5^, and it is even suggested that HIF2α may act as a renal cancer oncogene^6^. The binding of HIF1 and HIF2 factors to the hypoxia-responsive element (HRE) and the attachment of several cofactors triggers the transcription of several genes whose protein products are responsible for maintaining oxygen balance and facilitating cellular adaptation to hypoxia. HIFs are estimated to be responsible for the regulation of more than 100 genes leading to enhanced erythropoiesis, regulations of resistance to chemotherapeutics, or effects on extracellular matrix metabolism^7^. HIF1α plays an important role in cell death, and inflammation^8^, while HIF2α is a major regulator of erythropoietin production and vessel remodeling^9^. Recent studies indicate that HIF1α promotes fibrotic changes through vascular remodeling and angiogenesis. Vascular endothelial growth factor (VEGF) is the predominant HIF1α and HIF2α regulated proangiogenic factor in hypoxia-related diseases^10^. Hypoxia is frequently observed during tumor progression, which, in turn, leads to cancer cells starting to secrete immunosuppressive cytokines such as VEGF and transforming growth factor β (TGF-β)^11,12^. High levels of TGF-β1 as a response to tissue damage have been found to be strongly correlated with the development of connective tissues in the affected organs due to the production of collagen and subsequent fibrosis in tissues such as the kidneys or lungs^13,14^.

One of the problems in medical parasitology is opportunistic facultative organisms, including *Acanthamoeba* spp., which can have both free-living and parasitic lifestyles. Research on *Acanthamoeba* spp. concerns the brain, lungs, and eye cornea, which are the main biotopes of these amoebae^15^. However, recent analyses indicate that amoebae can also infect the kidneys, mainly in immunosuppressed hosts^16,17^. Previous studies showed a statistically significant increase in the level of kidney injury molecule 1 (KIM-1) and a statistically significant decrease in neutrophil gelatinase-associated lipocalin (NGAL) in immunocompetent mice infected with amoebae compared to mice from the control group. In mice with a reduced level of immune response infected with *Acanthamoeba* sp., statistically significant higher levels of KIM-1 and monocyte chemotactic factor 1 (MCP-1) were observed compared to uninfected mice^16^. One of the mechanisms causing the KIM-1 and MCP-1 increase is hypoxia. It was found that hypoxia induces the expression of KIM-1^18^ and MCP-1 by activating the renin-angiotensin system^19^. Additionally, the renal tubules with high levels of KIM-1 are surrounded by inflammatory cells and show signs of fibrosis^20^. In preliminary studies, lighter staining of the nuclei and cytoplasm of tubular cells, as well as infiltration of inflammatory cells in the cortical part of the kidneys of mice infected with *Acanthamoeba* sp. were observed; these changes were noted in the hosts in which an increase in KIM-1 levels was found^16,21^. Inflammatory cells and T lymphocytes can promote the expression of TGF-β, which induces the transformation of renal tubular cells into proliferating fibroblasts, causing fibrous changes in the renal parenchyma^22^. Such changes may be visible in the histological preparation in the form of a lighter colour of the kidney parenchyma. However, additional histological and immunohistochemical studies are necessary to confirm these changes.

The aim of the study was to determine the concentration and expression of HIF1α, HIF2α, and TGF-β1 in the kidneys of mice with different immune status infected with *Acanthamoeba* sp. Additionally, Masson trichrome staining was performed to detect collagen fibers, while angiogenesis was performed by assessing immunoexpression of VEGF and CD31 in the kidneys of hosts infected with *Acanthamoeba* sp. To our knowledge, this is the first paper concerning hypoxia-related mechanisms in the hosts with acanthamoebiasis.

## Material and methods

### Ethics statement

The experimental study on animals was carried out in 2016, and the necessary consents from the Local Ethical Committee in Szczecin (Resolution No. 29/2015 of June 22, 2015) and Poznań (Resolution No. 64/2016 of September 9, 2016) to conduct experiments on laboratory animals have been obtained. All animal experiments were performed in strict agreement with good animal practice with the recommendations in the Guide for Care and Use of Laboratory Animals and ARRIVE guidelines.

### Animal model

BALB/c strain mice (n = 96; mean weight 23 g) were obtained from a licensed breeder—the Centre of Experimental Medicine, Medical University in Bialystok, Poland. They were approximately 6–10 weeks old and had health certificates issued by a veterinarian. The animals were divided into four groups: (i) infected with *Acanthamoeba* sp., immunocompetent (group A, n = 30); (ii) infected with *Acanthamoeba* sp., immunosuppressed (AS group, n = 30); (iii) uninfected, immunocompetent (group C, n = 18); (iv) uninfected, immunosuppressed (CS group, n = 18). Four days before amoebae infection, mice from the AS and CS groups received methylprednisolone intraperitoneally. Then, mice from groups A and AS received a suspension of amoebae (10–20 thousand trophozoites) of the AM22 strain (GenBank: GQ342607.1), which came from a hemato-oncology patient^23^. Animals from the C and CS groups were administered the same volume of physiological saline. On days 8, 16, and 24 after infection (dpi), the animals were anesthetized with sodium pentobarbital (Euthasol vet, FATRO, Raamsdonksveer, the Netherlands; 2 ml/kg body weight) and dissected. Sterile kidneys were collected from the animals and preserved for further analysis. Samples for biochemical and molecular analysis were stored at −80 °C and those for histological analyses were fixed in paraformaldehyde and embedded into paraffin.

Analyses were performed on six mice from each group at each of the three-time points. Only the kidneys of animals in which amoebae were reisolated were used for the study. The detailed experimental design is presented in the works of Łanocha-Arendarczyk et al.^24^ and Kot et al.^16,17^. In the paper, we present analyses that use already published data. The animal model described here is the very same than the one presented in our previous papers^16,17^. Therefore, direct comparisons could be made between the present data and the previous ones.

### Analyses of HIF1α, HIF2α, TGF-β1 concentration by ELISA method

Analyses of HIF1α and HIF2α concentrations were performed by ELISA using Mouse HIF1α (Hypoxia-inducible factor 1-alpha) ELISA Kit (Fine tests, Wuhan, China; CAT no. EM0310) and HIF2α (Hypoxia-inducible factor 2-alpha) ELISA Kit (Fine tests, Wuhan, China; CAT no. EM1615), respectively. The concentration of TGF-β1 was also analyzed by ELISA method using Mouse TGF beta 1 ELISA Kit (Invitrogen, ThermoFisher, CA, USA; CAT no. BMS608-4). The material was prepared and tested according to the manufacturer’s recommendations. Measurement was performed on the EZ Read 2000, Biochrom Ltd., Cambridge, UK.

### Analyses of HIF1α, HIF2α, TGF-β1 gene expression by qRT-PCR

Quantitative mRNA expression of HIF1α and HIF2α genes was performed by real-time quantitative PCR. The relative expression of the studied genes was determined in relation to the average expression of *Gapdh* and *Beta-2 microglobulin*, reference genes with constitutive expression (housekeeping genes). Total RNA was isolated from kidney fragments stored at − 80 °C using the RNeasy MiniKit (Qiagen, Hilden, Germany) according to the manufacturer’s instructions. The concentration and purity of the isolated RNA were determined using a Nanodrop ND-1000 spectrophotometer (Thermo Fisher ScientificTM, Waltham, MA, USA). 1 μg of total RNA isolated from tissues was used for cDNA synthesis. The resulting template was transcribed into cDNA using a high-capacity reverse transcription kit (Thermo Fisher ScientificTM, Waltham, MA, USA; CAT no. K1612) with universal primers according to the manufacturer’s instructions. Gene expression analysis was performed using a 7500 Fast Real-Time PCR system (Applied Biosystems, Foster City, California, USA) with Power SYBR Green PCR Master Mix reagent (Applied Biosystems, Foster City, CA, USA; CAT no. A25918). The following primer pairs were used: *Gapdh* forward: GGA GAA ACC TGC CAA GTA TGA TG, reverse: GAC AAC CTG GTC CTC AGT GTA GC; *Beta-2 microglobulin* forward: CAT ACG CCT GCA GAG TTA AGC A, reverse: GAT CAC ATG TCT CGA TCC CAG TAG; HIF1α forward: GTC CCA GCT ACG AAG TTA CAG C, reverse: CAG TGC AGG ATA CAC AAG GTT T; HIF2α forward: GAG GAA GGA GAA ATC CCG TGA, reverse: TAT GTG TCC GAA GGA AGC TGA; TGF-β1 forward: CCA CCT GCA AGA CCA TCG AC, reverse: CTG GCG AGC CTT AGT TTG GAC.

### Analyses of HIF1α, HIF2α, CD31, and VEGF immunoexpression by IHC method

In order to expose the epitopes for the IHC procedure, the deparaffinized and rehydrated sections were boiled in Target Retrieval Solution (Dako, Glostrup, Denmark, CAT no. S2368) at pH 6.0 for 30 min. Once cooled and washed with PBS, the endogenous peroxidase was blocked by using a peroxidase-blocking solution (Dako, Glostrup, Denmark, CAT no. S2023) for 10 min, and then the slides were incubated in a humid chamber for 30 min with primary antibodies: multiclonal anti-CD31 (Abcam, Cambridge, UK, CAT no. ab281583; final dilution 1:100), monoclonal anti-VEGF (ThermoFisher, CAT no. SP-07-01; final dilution 1:50), polyclonal anti-HIF1alpha (Abcam, Cambridge, UK, CAT no. ab228649; final dilution 1:100), and polyclonal anti-HIF2alpha (antibodies.com, Stockholm, Sweden, CAT no. a10163, final dilution 1:100) in antibody diluent with background-reducing components (Dako, Glostrup, Denmark, CAT no. S0809). Next, the sections were incubated with a complex containing a secondary antibody conjugated with horseradish peroxidase (Dako, Glostrup, Denmark, CAT no. K8002). To visualize the antigen–antibody complexes, an EnVision FLEX system was used (Dako, Glostrup, Denmark, CAT no. K8007) based on the reaction of horseradish peroxidase with DAB as a chromogen, according to the included staining procedure instructions. Sections were washed in distilled H_2_O and counterstained with Mayer’s hematoxylin (Sigma-Aldrich Co., St Louis, MO, USA). For the negative control, specimens were processed in the absence of primary antibodies. Positive staining was determined microscopically (Olympus BX 41, Hamburg, Germany) through visual identification of brown pigmentation.

### Masson’s trichrome staining for collagen analysis

Kidney sections were deparaffinized and rehydrated. They were stained in modified Weigert’s iron hematoxylin and washed in distilled water. Next, they were stained in 1% acid fuchsin solution (Sigma-Aldrich, St. Louis, MO, USA) in distilled water and subsequently in 5% phosphotungstic acid solution. Next, the kidney sections were stained in a solution of 1% aniline blue (Sigma-Aldrich, St. Louis, MO, USA), rinsed in distilled water, and differentiated in 1% acetic acid solution. Afterward, the slides were dehydrated and coverslipped in a mounting medium.

### Quantitative analysis of IHC and collagen

All slides obtained under the immunostaining of VEGF, CD31, HIF1α, and HIF2α and also under Masson trichrome staining were scanned at 400× magnification (resolution of 0.25 μm/pixel) by using a ScanScope AT2 scanner. In order to minimize focus problems, the scanner was preconfigured. The ImageScope viewer software (Aperio Technologies, Inc., Vista, CA, USA) provided digital images for examination on a computer screen.

For the quantitative analysis of CD31 expression in the particular layers of the kidneys, the *microvessel analysis v1* algorithm (Aperio Technologies, Inc., Vista, CA, USA) was used. The microvessel density (number of vessels per unit area), mean vessel area, and mean vessel perimeter were calculated. For the analysis of VEGF, HIF1α, and HIF2α *cytoplasmic v9* algorithm was used. The percentages of VEGF, HIF1α, and HIF2α-positive cells with weak, moderate, and strong immunostaining were determined. The algorithm automatically classified the staining as weak, moderate, or strong depending on the optical density ranges for a given level of immunoexpression. For the analysis of collagen deposition (slides stained with Masson trichrome), a *positive pixel count v9* algorithm was used.

The areas of all quantitative analysis were manually determined. The percentage of positive cells for all analyses was counted in a total of 60 high-power random fields with an average area of 3.94‒14.72 mm^2^ (5 per group). The quantitative data for CD31 were assessed separately for the cortex and medulla.

### Statistical analysis

The obtained results were subjected to statistical analysis using the Statistica Stat Soft and GraphPad 4.0 programs. The arithmetic mean (AM) and standard deviation (SD) from AM were calculated. The compliance of the distributions of the obtained results with the expected normal distribution was examined using the Shapiro–Wilk test. The data were statistically analyzed using non-parametric tests: for two variables—the Mann–Whitney test (U), and for three variables the Kruskall-Wallis test (H) followed by Dunn’s post hoc test. Additionally, some correlations were performed on the basis of Spearman’s rank correlation factor.

## Results

### HIF1α and HIF2α

#### Gene expression of HIF1α and HIF2α

**Fig. 1:**
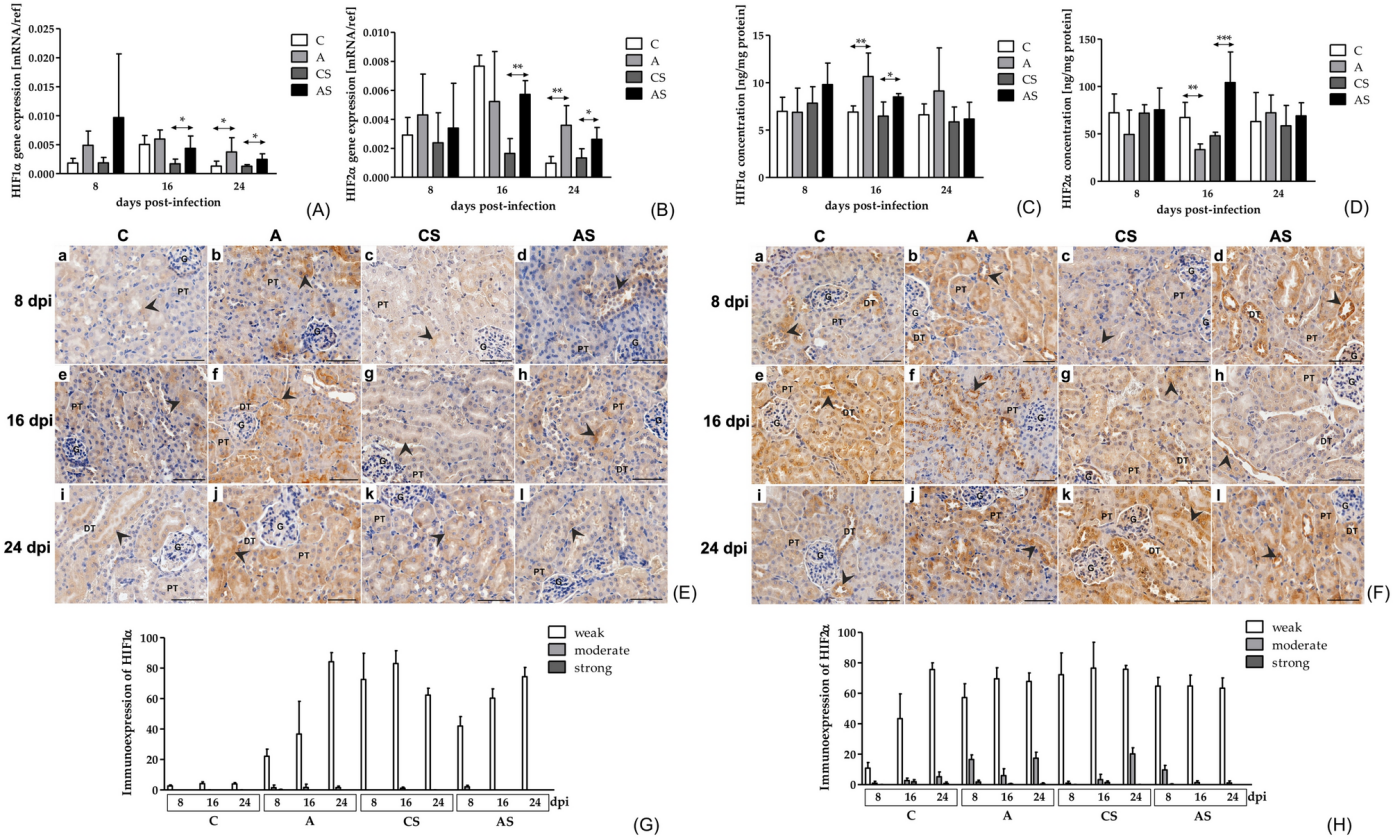
**(A–B)** The gene expression HIF1α (**A**) and HIF2α (**B**) in the kidneys of mice infected with *Acanthamoeba* sp. HIFs were determined using qRT-PCR. Data represent the means and standard deviations for six experiments. * statistically significant differences *p* < 0.05, ** statistically significant differences *p* < 0.01, Mann–Whitney U test **(C–D)** The concentration of HIF1α (**C**) and HIF2α (**D**) in the kidneys of mice infected with *Acanthamoeba* sp. HIFs were determined using ELISA method. Data represent the means and standard deviations for six experiments, * statistically significant differences *p* < 0.05, ** statistically significant differences p < 0.01, *** statistically significant differences *p* < 0.001, Mann- Whitney U test **(E–F)** Representative light micrographs showing HIF1α (**E**) and HIF2α (**F**) immunoexpression (brown-stained cytoplasm; black arrowheads) in renal tubular epithelial cells of kidney cortex in the control mice (a, e, i), immunocompetent mice infected with *Acanthamoeba* sp. (b, f, j), immunosuppressed control mice (c, g, k) and immunosuppressed mice infected with *Acanthamoeba* sp. (d, h, l) after 8 (a‒d), 16 (e‒h) and 24 (i‒l) days post infection. Scale bar—50 μm. G—glomerulus, PT—proximal tubule, DT—distal tubule **(G–H)** The immunoexpression HIF1α (**G**) and HIF2α (**H**) in the kidneys of mice infected with *Acanthamoeba* sp. Data represent the means and standard deviations for six experiments. A ‒ immunocompetent mice infected with *Acanthamoeba* sp., AS—immunosuppressed mice infected with *Acanthamoeba* sp., C—immunocompetent control mice, CS—immunosuppressed control mice.

The mRNA levels of HIF1α in the kidneys of immunocompetent mice were at a similar level at different time points. A statistically significant difference between the A and C groups was noted at 24 dpi; a higher mRNA level was found in the A group (U = 3.00, *p* = 0.02; Fig. 1A). Gene expression of HIF1α in immunosuppressed mice was not significant between time points. There was a difference between the AS and CS groups at 16 as well as 24 dpi; the mRNA level of HIF1α was higher in the AS group on each of these days (16 dpi: U = 4.00, *p* = 0.03; 24 dpi: U = 2.00, *p* = 0.02; Fig. 1A).

Gene expression of HIF2α in immunocompetent mice did not differ between 8, 16, and 24 dpi. A statistically significant difference between the A and C groups was noted at 24 dpi; a higher mRNA level was found in the A group (U = 0.00, *p* = 0.002; Fig. 1B). mRNA level of HIF2α in immunosuppressed mice also did not differ at studied time points. There were differences in the mRNA level of HIF2α between AS and CS groups at 16 as well as 24 dpi (16 dpi: U = 3.00, *p* = 0.004; 24 dpi: U = 2.00, *p* = 0.02; Fig. 1B).

#### Concentration of HIF1α and HIF2α

The concentration of HIF1α in immunocompetent mice was not statistically different between 8 dpi vs. 16 dpi vs. 24 dpi. A statistically significant higher concentration of HIF1α was observed in the A group compared to the C group at 16 dpi (U = 0.00, *p* = 0.002; Fig. 1C). HIF1α levels in immunosuppressed mice decreased at each time point (H = 8.33, *p* = 0.02). Further analysis with Dunn’s test showed difference between 24 dpi compared to 8 dpi. A statistically significant higher concentration of HIF1α was found in the kidneys of mice from the AS group compared to the CS group at 16 dpi (U = 4.00, *p* = 0.03; Fig. 1C). Taking into account the immune status of the hosts, there were no significant differences between A and AS groups.

The concentration of HIF2α in the immunocompetent mice decreased at 16 dpi and then increased at 24 dpi (H = 7.24, *p* = 0.03). Further analysis with Dunn’s test showed difference between 24 dpi compared to 16 dpi. A statistically significantly lower concentration of HIF2α was noted in the A group at 16 dpi compared to mice from the control group (U = 0.00, *p* < 0.001; Fig. 1D). The level of HIF2α in the immunosuppressed mice did not differ between 8 dpi vs. 16 dpi vs. 24 dpi. There was a statistically significant difference between AS and CS only at 16 dpi; a higher concentration was found in the kidneys of mice infected with *Acanthamoeba* sp. (U = 0.00, *p* < 0.001; Fig. 1D). Taking into account the immune status of the hosts, a higher concentration of HIF2α was found in the AS group at 8 and 16 dpi (8 dpi: U = 16.00, *p* = 0.03; 16 dpi: U = 0.00, *p* < 0.001).

#### Immunoexpression of HIF1α and HIF2α

Immunoexpression of HIF1α was observed predominantly in the cytoplasm of proximal and distal tubular epithelial cells in the cortex and collecting tubule epithelial cells in the medulla (Fig. 1E). Immunoexpression varied depending on the kidney part. The strongest HIF1α immunoexpression was usually found in the inner medulla.

In uninfected immunocompetent mice, only weak and moderate immunoexpression was noted. However, in mice from the A group, we observed weak, moderate, and strong immunoexpression of HIF1α. Statistically significant differences were found in renal cells with weak and moderate immunoexpression between A and C groups on each studied day (U = 0.0, *p* = 0.008). In immunosuppressed mice infected and uninfected with the protozoan, kidney cells showed weak as well as moderate immunoexpression of HIF1α. Interestingly, renal cells in mice from the CS group at 8 dpi and from the AS group at 24 dpi showed only weak immunoexpression. Statistically significant differences were found between cells with weak immunoexpression between AS and CS groups on each studied day (U = 0.0, *p* = 0.008 for 8, 16 dpi and U = 1.0, *p* = 0.02 for 24 dpi). Moreover, we found statistically significant differences between renal cells with moderate expression between AS and CS groups at 8 and 16 dpi (U = 0.0, *p* = 0.008; Fig. 1G).

HIF2α immunoexpression was also observed in the cytoplasm of proximal and distal tubular epithelial cells in the cortex and collecting tubular epithelial cells in the medulla (Fig. 1F). The strongest HIF2α immunoexpression was noted in the outer part of the medulla. Moreover, HIF2α immunoexpression was demonstrated in cortical and medullary endothelial cells.

Weak, moderate, and strong HIF2α immunoexpression were found in uninfected and amoeba-infected immunocompetent mice. Statistically significant differences were found in cells with weak expression between A and C groups on each studied day (U = 0.0, *p* = 0.008 for 8 and 16 dpi, U = 2.0, *p* = 0.03 for 24 dpi). Significant differences were also found in cells with moderate expression of HIF2α between A and C groups at 8 and 24 dpi (U = 0.0, *p* = 0.008) and in cells with strong expression between A and C groups at 8 dpi (U = 0.0, *p* = 0.008). In immunosuppressed mice infected and uninfected with *Acanthamoeba* sp., kidney cells had weak, moderate, and strong immunoexpression of HIF2α. Interestingly, renal cells in mice from the AS group at 16 and 24 dpi showed only weak and moderate HIF2α expression. A significant difference was observed in cells with weak expression between AS and CS groups at 8 dpi (U = 0.0, *p* = 0.008). Moreover, we found differences in cells z moderate expression between AS and CS groups at 8 and 24 dpi (U = 0.0, *p* = 0.008) and in cells with strong expression between AS and CS groups at 16 and 24 dpi (U = 0.0, *p* = 0.008; Fig. 1H).

### TGF-β1

#### Gene expression of TGF-β1

**Fig. 2:**
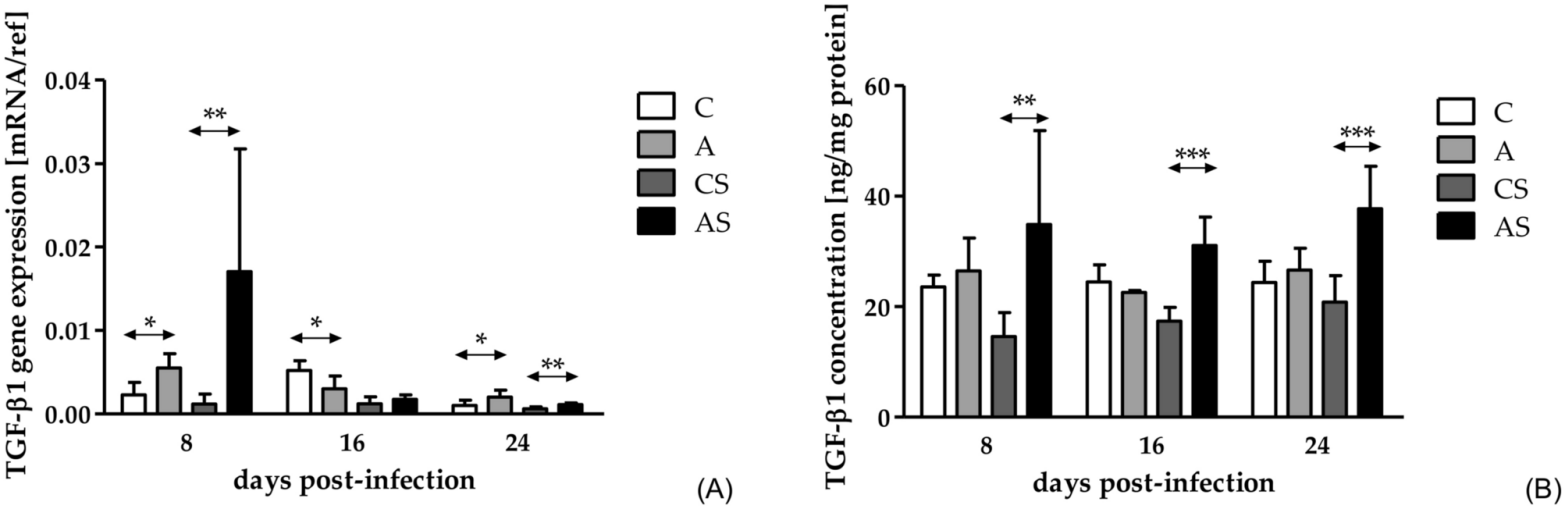
The gene expression **(A)** and concentration **(B)** of TGF-β1 in the kidneys of mice infected with *Acanthamoeba* sp. TGF-β1 were determined using qRT-PCR and ELISA methods. Data represent the means and standard deviations for six experiments. A—immunocompetent mice infected with *Acanthamoeba* sp., AS—immunosuppressed mice infected with *Acanthamoeba* sp., C—immunocompetent control mice, CS—immunosuppressed control mice, * statistically significant differences *p* < 0.05, Mann–Whitney U test.

The mRNA level of TGF-β1 in the immunocompetent mice decreased at each time point (H = 9.44, *p* = 0.009). Further analysis with Dunn’s test showed difference between 24 dpi compared to 8 dpi. Statistically significant differences between the A and C groups were observed at 8 (U = 1.00, *p* = 0.02), 16 (U = 2.00, *p* = 0008), and 24 dpi (U = 4.00, *p* = 0.03; Fig. 2A).

Gene expression of TGF-β1 in the immunosuppressed mice also decreased at each time point (H = 10.93, *p* = 0.004). Analysis with Dunn’s test showed statistical difference between 24 dpi compared to 8 dpi. Interestingly, the difference between the gene expression at 8 dpi and 16 dpi was an order of magnitude. Moreover, there were differences between the AS and CS groups at 8 and 24 dpi; on each studied day, the mRNA level of TGF-β1 in the AS was higher (8 dpi: U = 4.00, *p* = 0.03; 24 dpi: U = 2.00, *p* = 0.008; Fig. 2A).

#### Concentration of TGF-β1

The concentration of TGF-β1 in the immunocompetent mice increased at 16 dpi compared to 8 dpi and then decreased at 24 dpi (H = 9.46, *p* = 0.009). Further analysis with Dunn’s test showed difference between 24 dpi compared to 16 dpi. There were no statistically significant differences between A and C groups.

The level of TGF-β1 in the immunosuppressed mice decreased at 16 dpi and then increased at 24 dpi, but the differences were not statistically significant. Higher concentrations of TGF-β1 were observed in the AS group at 8, 16, and 24 dpi compared to the CS group (8 dpi: U = 4.00, *p* = 0.001; 16 dpi: U = 0.00, *p* < 0.001; 24 dpi: U = 4.00, *p* < 0.001; Fig. 2B**)**.

Taking into account the immunological status of the hosts, there were higher TGF-β1 concentrations in the AS group at 16 (U = 0.00, *p* < 0.001) and 24 dpi (U = 8.00, *p* = 0.003) than in the A group.

### Analysis of collagen deposition

**Fig. 3:**
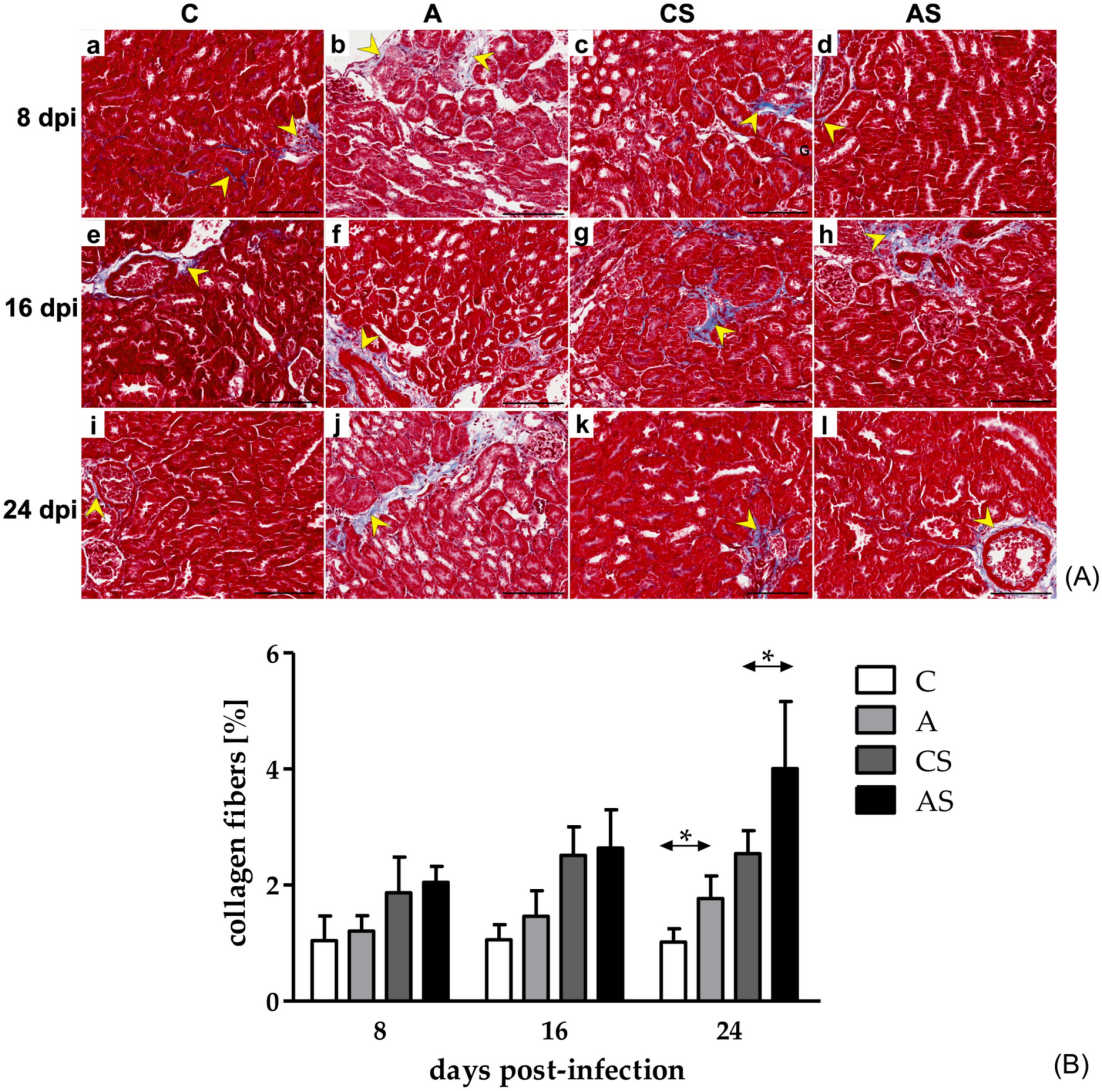
**A** Representative light micrographs of Masson trichome-stained (blue-stained collagen fibers; yellow arrowheads) kidney in the control mice (a, e, i), immunocompetent mice infected with *Acanthamoeba* sp. (b, f, j), immunosuppressed control mice (c, g, k) and immunosuppressed mice infected with *Acanthamoeba* sp. (d, h, l) after 8 (a‒d), 16 (e‒h) and 24 (i‒l) days post infection. Scale bar—50 μm. **(B)** The percentage of collagen in the kidneys of mice infected with *Acanthamoeba* sp. Data represent the means and standard deviations for six experiments. A—immunocompetent mice infected with *Acanthamoeba* sp., AS—immunosuppressed mice infected with *Acanthamoeba* sp., C—immunocompetent control mice, CS—immunosuppressed control mice, * statistically significant differences *p* < 0.05, Mann–Whitney U test.

Masson trichrome-stained collagen fibers were observed predominantly in the renal interstitium, and predominantly in the connective tissue septa surrounding blood vessels (Fig. 3A).

The percentage of collagen in all studied groups of mice was not higher than 6%. Nevertheless, we observed some statistically significant differences. There was significantly higher percentage of collagen in the A group compared to the C group at 24 dpi (U = 1.00, *p* = 0.02; Fig. 3B). In the AS group at 24 dpi, there was also higher percentage of collagen than in the CS group (U = 2.0, *p* = 0.03; Fig. 3B). Comparing the immunological status of the host, the differences were observed between the A and AS groups at 8 (U = 0.0, *p* = 0.008), 16 (U = 2.0, *p* = 0.03), and 24 dpi (U = 0.0, *p* = 0.008).

Additionally, the correlations between the percentage of collagen fibers and the levels of KIM-1 in the kidneys of *Acanthamoeba* spp.—infected mice were analysed. There were no statistically significant relationships in the studied groups, neither in the infected nor in the uninfected animals. Detailed data is presented in Table S1 in the supplementary materials.

### Vessel parameters for CD31

**Fig. 4:**
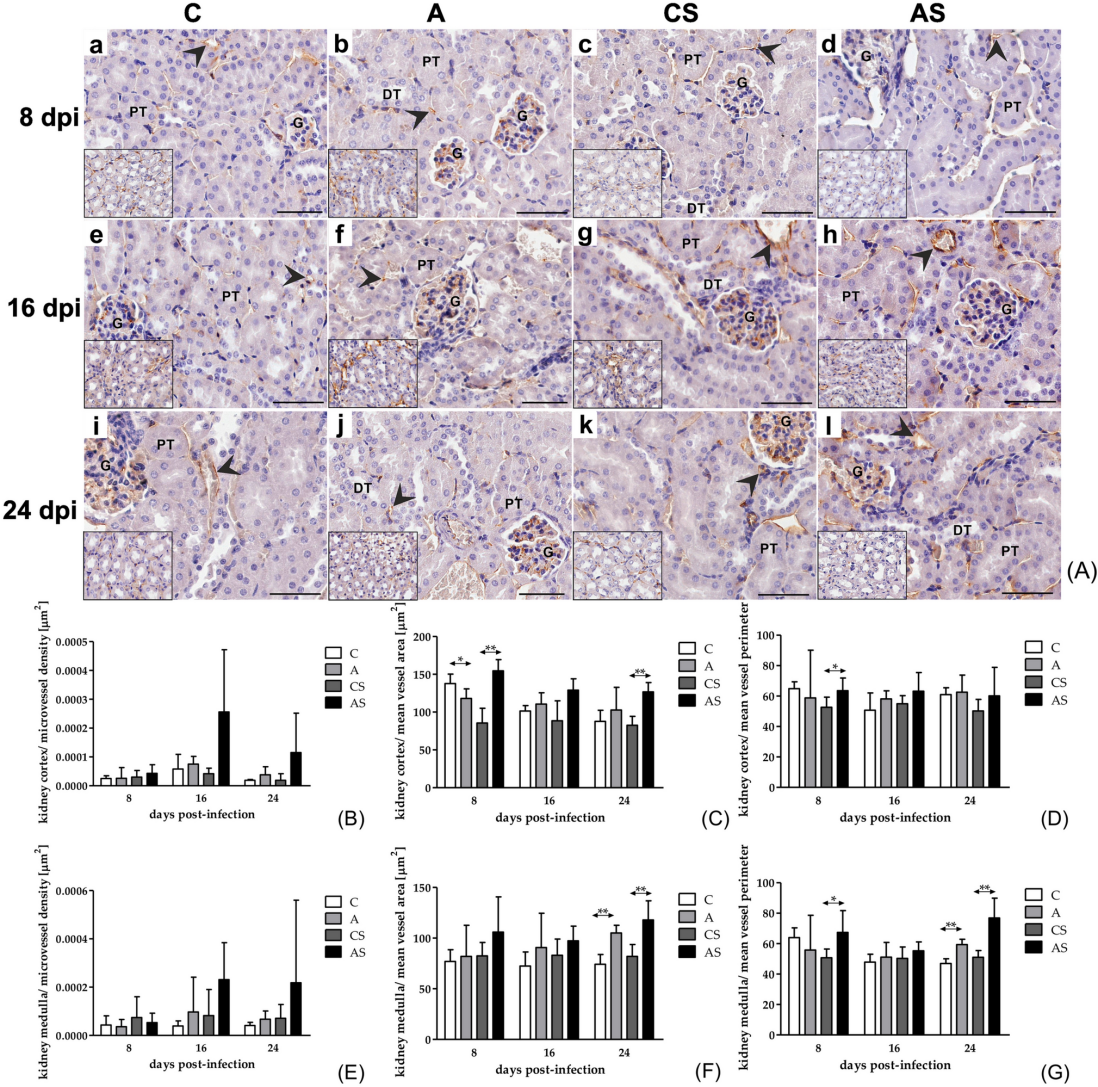
**(A)** Representative light micrographs showing CD31 immunoexpression (brown-stained cytoplasm; black arrowheads) in the glomerular endothelium and in endothelial cells of interstitial vessels of kidney cortex in the control mice (a, e, i), immunocompetent mice infected with *Acanthamoeba* sp. (b, f, j), immunosuppressed control mice (c, g, k) and immunosuppressed mice infected with *Acanthamoeba* sp. (d, h, l) after 8 (a‒d), 16 (e‒h) and 24 (i‒l) days post infection. The insets show a renal medulla. Scale bar ‒ 50 μm. DT, distal tubule; G, glomerulus; PT, proximal tubule; **(B–G)** Microvessel density (B,E), mean vessel area (**C**, **F**) and mean vessel perimeter (**D**, **G**) for CD31 in the kidney cortex (**B**, **C**, **D**) and medulla (**E**, **F**, **G**) of mice infected with *Acanthamoeba* sp. Data represent the means and standard deviations for six experiments. A—immunocompetent mice infected with *Acanthamoeba* sp., AS—immunosuppressed mice infected with *Acanthamoeba* sp., C—immunocompetent control mice, CS—immunosuppressed control mice, *statistically significant differences *p* < 0.05; ** statistically significant differences *p* < 0.01, Mann–Whitney U test.

CD31 immunoexpression was observed in the glomerular endothelium and the interstitial vessels of the kidney in all groups (Fig. 4A).

In the kidney cortex, there was no statistical significance in microvessel density (number of vessels per unit area) or mean vessel perimeter between the A and C groups (Fig. 4B, D, respectively). A significantly lower (U = 0.0, *p* < 0.001) mean vessel area was observed for CD31 in the A group compared to control mice at 8 dpi (Fig. 4B). In the kidney medulla, there was no statistical significance in microvessel density between A and C groups (Fig. 4E). In the 24^th^ day post-infection, there was a significantly higher vessel area (U = 0.0, *p* = 0.008; Fig. 4F) and mean vessel perimeter (U = 0.0, *p* = 0.008; Fig. 4G) in A group compared to control group.

In the immunosuppressed mice, we observed a significantly higher vessel area for CD31 in the AS group compared to the CS group at 8 dpi (U = 0.0, *p* < 0.001, Fig. 4C). On the 8^th^ day post-infection, we also observed a significantly higher mean vessel perimeter for CD31 between AS vs CS groups (U = 7.0, *p* = 0.03; Fig. 4D). We noted higher vessel area for CD31 (U = 0.0, *p* = 0.008) in the AS group compared to CS group at 24 dpi (Fig. 4C). In the kidney medulla, there was no statistical significance in microvessel density between AS and CS groups (Fig. 4E). In 8th day post-infection, we also observed a significantly higher mean vessel perimeter for CD31 between AS vs CS groups (U = 6.0, *p* = 0.02; Fig. 4G). While in 24th day, there was higher mean vessel area (Fig. 4F) and mean vessel perimeter (U = 0.0, *p* = 0.008; Fig. 4G) in AS group compared to control mice.

In the kidney cortex, we observed statistical significance in microvessel density for CD31 in the immunocompetent infected mice between 8 dpi vs. 16 dpi vs 24 dpi (H = 7.43, *p* = 0.02). Further analysis with Dunn’s test showed difference between 16 dpi compared to 8 dpi. In the kidney medulla, there was only statistical significance in mean vessel perimeter in the immunosuppressed infected mice between 8 dpi vs. 16 dpi vs 24 dpi. (H = 6.48, *p* = 0.04) (Fig. 4G). Analysis with Dunn’s test showed difference between 24 dpi compared to 16 dpi.

### Immunoexpression of VEGF

**Fig. 5:**
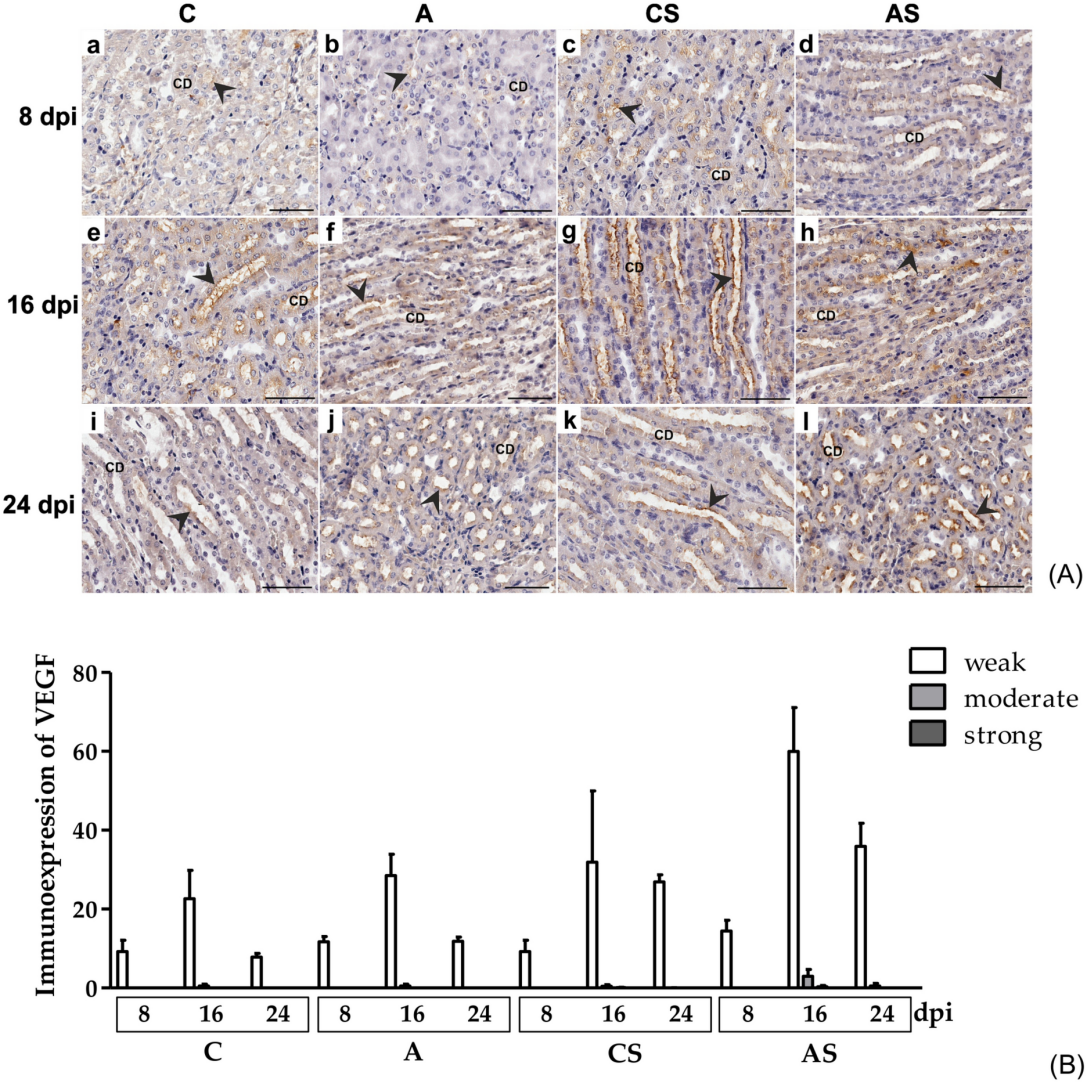
(**A**) Representative light micrographs showing VEGF immunoexpression (brown-stained cytoplasm; black arrowheads) in the outer medulla collecting duct of kidney in the control mice (a, e, i), immunocompetent mice infected with *Acanthamoeba* sp. (b, f, j), immunosuppressed control mice (c, g, k) and immunosuppressed mice infected with *Acanthamoeba* sp. (d, h, l) after 8 (a‒d), 16 (e‒h) and 24 (i‒l) days post infection. Scale bar—50 μm. CD—collecting duct; (**B**) Immunoexpression of VEGF in the kidneys of mice infected with *Acanthamoeba* sp. Data represent the means and standard deviations for six experiments. A—immunocompetent mice infected with *Acanthamoeba* sp., AS—immunosuppressed mice infected with *Acanthamoeba* sp., C—immunocompetent control mice, CS—immunosuppressed control mice, dpi—days post infection.

VEGF immunoexpression was observed predominantly in the cytoplasm of collecting tubule epithelial cells in the outer medulla in the kidneys of all groups (Fig. 5A). Moreover, weak immunoexpression of VEGF was sometimes visible in proximal and distal tubules in the renal cortex.

The renal cells in the immunocompetent uninfected and *Acanthamoeba* sp.-infected mice showed weak and moderate VEGF immunoexpression; it is worth noting that moderate expression was observed only in mice at 16 dpi. A statistically significant difference was noted in cells with weak VEGF immunoexpression between A and C groups in long-lasting infection (U = 0.0, *p* = 0.008; Fig. 5B). In immunosuppressed mice (AS and CS groups), the kidney cells showed weak, moderate and strong immunoexpression of VEGF. Interestingly, strong expression was observed only on the 16^th^ day of infection. We found differences in cells with weak immunoexpression between AS and CS groups at 8 and 24 dpi (U = 2.0, *p* = 0.03). There were no differences between cells with moderate and strong expression of VEGF between AS and CS groups (Fig. 5B).

## Discussion

Acanthamoebiasis pathogenesis is not well understood but most probably it is the result of the dynamic interaction of *Acanthamoeba* spp. and their virulence factors with different components of the immune system. Hypoxia plays an important role in infectious diseases, leading to changes in cellular metabolism that may contribute to or protect against pathophysiology. Due to specific tissue tropisms, many parasites exhibit a certain oxygen affinity. But oxygen is also one of the main host stressors that influence the parasite’s survival and pathogenicity. *Acanthamoeba* spp. similar to *Entamoeba histolytica* can upregulate their antioxidant machinery, which includes superoxide dismutase and thioredoxin to evade host immune defense^25,26^. In our previous study, *Acanthamoeba* sp. lead to increased levels of NADPH oxidase 2 and 4 (NOX2 and NOX4, respectively) in the kidneys of immunocompetent and immunosuppressed hosts^17^. NOXs are enzymes generating reactive oxygen species (ROS) that regulate the activity of cellular transduction pathways and the associated mechanisms of modulation of the HIF1 pathway. Of the free radicals that activate HIF1, the most important role is played by hydrogen peroxide, which is formed by NOXs^27,28^.

The renal tissue is very sensitive to hypoxia and has been known as a significant factor in the pathogenesis of kidney diseases^29^. In our novel study, we analyzed HIF1α and HIF2α by molecular, immunoenzymatic as well as immunohistochemistry methods in the kidneys of hosts with systemic acanthamoebiasis. *Acanthamoeba* sp. induces hypoxia in mice with normal and reduced immune response by increasing gene and/or protein expression of HIF1α as well as HIF2α. Among parasitic diseases, the upregulation of HIF1α mRNA expression was also observed in the renal tissue of hosts with malaria^30,31^. Elias et al.^30^ found an increase of tissue hypoxia in the kidneys of infected animals. Alterations to HIF1α expression and hypoxic-related signaling have been described during other protozoan parasitic infections, such as amebiasis, toxoplasmosis, and leishmaniasis^25^. It is worth noting that the activation of HIF1α in host cells affected by *Toxoplasma gondii* promotes the survival and growth of this protozoan parasite albeit its exact mechanisms are not known^32^. The study on *Leishmania donovani* also confirmed that HIF1α promotes survival of parasites in host macrophages by downregulating NF-κB mediated proinflammatory immune response^33,34^. On the contrary, Werth et al.^35^ found that HIF1 plays an important role in the host defence against various pathogens. Alonso et al.^36^ reported that activation of the HIF1α/MIF axis via NOX2/ROS induction promotes killing of *L. amazonensis* amastigotes by macrophages. Decreased oxygen tension is known to induce the production of HIF1α that requires further stabilization by NOX2-dependent ROS activity^25,37–39^. Based on our previous^17^ and presented papers, we found that the mRNA NOX2 gene was elevated on the same days as the mRNA of HIF1α and HIF2α. These results establish a mechanism linking HIFs and ROS generation in the kidneys in response to *Acanthamoeba* sp. We speculate that the activation of HIF1α and/or HIF2α in the kidneys of hosts with acanthamoebiasis might also be induced via NOX2/ROS. But more direct evidence is needed to confirm this suggestion. It is also worth noting, that we reisolated the amoebae from the fragment of kidney samples of mice on NN agar but we did not observe any developmental forms of amoebae in the histological slides of the kidneys ^21^. The question still remains whether HIF activation by *Acanthamoeba* sp. favour the pathogen, the host, or both. Parasites use different strategies to evade innate host defenses, including hijacking the host’s cellular signaling pathways and transcription factors^40^. Understanding these pathways could provide a better appreciation of the complexity of the diseases.

In this study, we also observed a significantly higher proportion of HIF2α than HIF1α. These factors affect disease development and progression differently. Werth et al.^35^ showed HIF1 activation in infections with human pathogenic microorganisms, while HIF2 activation was not observed. The Authors^35^ speculated that HIF1 correlates with the course of bacterial infection because acute infections are linked to a rapid and strong HIF1 activation whereas such activation is missing in chronic infections. Additionally, they suggest that overwhelming HIF1 activation might be detrimental in severe bacterial infections^35^. In cutaneous leishmaniasis, both HIF1α and HIF2α are active but it is suggested that hypoxia promotes macrophage phagocytosis in a HIF1α-dependent manner, while HIF2α acts as a phagocytic repressor during *Leishmania* infection^41^. Some studies also reported that HIF1α is responsible for the polarization towards the M2-like phenotype of tumor-associated macrophages, promoting tumor growth^42^. HIFs also differ in their activity under conditions of sudden and prolonged hypoxia. It has been found that in the tissue where tumor growth results in reduced oxygen availability, HIF1 is expressed first, and later, under conditions of chronic hypoxia HIF2 begins to play a major role. This switch in the regulation of gene expression, occurring in both directions, allows cells to precisely adapt to changing conditions^43^. The exact role of HIF1α and HIF2α in infectious diseases, including acanthamoebiasis, remains unknown.

Lack of oxygen is a significant factor influencing the formation of new blood vessels. There is then an increase in VEGF expression as a result of HIFs^44^. VEGF is a regulator of vessel permeability ^45^. The degree of angiogenesis is also assessed by morphological studies using antibodies against CD31. Assessment of angiogenesis is based on mean microvessel density, where the mean number of vessels/mm^2^ is determined, mean vessel area, and vessel perimeter. In malaria, HIF1 can induce a morphological modification in the kidneys such as changes in vascular permeability^46^. Elias et al.^30^ noted a downregulation of VEGF, while Park et al.^31^ observed increased expression of this angiogenic factor in the mouse kidneys with malaria. Additionally, the Authors found increased blood vessel formation (for CD31) in the kidneys of mice with malaria, in a parasitemia-dependent manner^31^. In our study, we did not observe a progressive increase of VEGF in the kidneys of hosts with systemic acanthamoebiasis; moderate and strong expression of the angiogenic factor was observed mainly in the immunosuppressed mice infected with *Acanthamoeba* sp. However, there was increased mean vessel area and vessel perimeter in the kidney medulla of immunocompetent mice with long-lasting acanthamoebiasis, as well as increased mean vessel area and vessel perimeter in the kidney cortex and medulla of immunosuppressed mice at 8 and 24 dpi. The results of the study suggest that *Acanthamoeba* sp. may cause tissue damage through HIF1α and HIF2α-dependent manner which in turn lead to angiogenesis in the kidneys of hosts.

Scientific data indicates that prolonged hypoxia and angiogenesis are closely associated with the progression of fibrosis^47,48^. The main profibrogenic cytokine is transforming growth factor β (TGF-β)^49^. It belongs to the cytokine group, which exerts multifunctional effects on cell proliferation, migration, differentiation, and apoptosis^50^. Significant relationships exist between the TGF-β expression and the degree of kidney fibrosis^51^. On the other hand, a number of studies have reported that TGF-β deficient mice suffered from lethal inflammation and early death, suggesting a protective role for TGF-β in renal inflammation. Thus, TGF-β may exert its diverse role in renal inflammation and fibrosis by interacting with many signaling pathways and molecules^52^. TGF-β was examined in the kidneys of animals infected with some parasitic protozoa. In the kidneys infected with *T. gondii,* Pereira et al.^53^ found increased immunoexpression of TGF-β and increased collagen deposition, which suggests that fibrosis is a consequence of increased TGF-β synthesis. The increased level of TGF-β was also observed in the kidneys of mice infected with *L. donovani*^54^ and in the kidneys of dogs infected with *L. infantum*^55^. Histological sections of dogs’ kidneys stained with Masson’s trichrome confirmed fibroblast proliferation and increased collagen deposition in the tubules and the interstitium of the renal cortex and renal medulla layer in comparison with controls^55^. In the kidneys of mice with systemic acanthamoebiasis, increased TGF-β gene expression and/or concentration was observed in the immunocompromised host at 8, 16, and 24 dpi. In the immunocompetent hosts, only gene expression of TGF-β was increased, and it was noted at 8 and 24 dpi. Collagen deposition in the kidneys of mice infected with *Acanthamoeba* sp. was observed in both immunocompetent and immunosuppressed hosts. An increased percentage of collagen was observed in prolonged acanthamoebiasis (24 dpi) in kidney hosts regardless of their immunological status. Cohen et al.^56^ quantified renal fibrosis by histological fibrosis scoring. Renal tissues were stained with Masson’s trichrome and the cortical fibrosis was scored based on a scale of 0 through 4, by assessment of microscopic fields showing stained collagen (0 = none, 1 = minimal, 2 = less than half of fields fibrotic, 3 = more than half of fields fibrotic, 4 = all fields fibrotic)^56^. Based on this scale, in the kidneys of mice infected with *Acanthamoeba* sp., fibrosis score was 0 or 1; and it was seen mostly near the vessel area. Additionally, to check the relevance of the observed collagen deposition, we analyzed the Spearman’s rank correlations between the percentage of collagen fibers and the concentration of kidney injury molecule 1 (KIM-1 levels are presented in^16^). All relationships both in the infected (A and AS) and uninfected (C and CS) groups were insignificant (*p* > 0.05). The deposition of collagen in the kidneys of mice with systemic acanthamoebiasis is not large and should not be called fibrosis, albeit it indicates some changes that occur in renal tissue during long-term infection. In future studies, it is worth checking urine collagen breakdown products, because they are sensitive early markers of interstitial fibrosis, preceding histological fibrotic changes^57^.

The biochemical pathways leading to collagen deposition are different. Verçosa et al.^47^ found that collagen fiber deposition in the kidneys of dogs with canine leishmaniasis does not occur via the canonical pathway of TGF-β, but rather through the expression of MCP-1. In our previous study^16^, we examined the concentration of MCP-1 in the kidneys of mice infected with *Acanthamoeba* sp. We observed a statistically higher concentration in the AS group compared to CS at 16 dpi, and a lower level in the AS group compared to CS at 24 dpi^16^. Therefore, we suspect that collagen deposition in the renal tissue of hosts with disseminated acanthamoebiasis occurs most probably via TGF-β.

Increased expression of TGF-β can also induce the apoptosis process^54,55^. Kumar et al.^54^ suggested that TGF-β may mediate apoptosis in the renal cells in the *Leishmania* spp.-infected hosts, which resulted in cellular disorganization and renal tissue damage. In our previous study^17^, we analyzed the apoptosis process in the kidneys of the same murine model. We found that in immunocompetent hosts, *Acanthamoeba* sp. do not lead to dysregulation of the Bax/Bcl-2 ratio, and in long-term infection, they even inhibit apoptosis of host renal cells. While in the kidneys of immunosuppressed hosts, infection with *Acanthamoeba* sp. leads to increased apoptosis by the intrinsic pathway^17^. Similar to the paper by Kumar et al.^54^, we suggest that TGF-β may mediate apoptosis in the renal cells in the *Acanthamoeba* sp.-infected hosts because TGF-β concentration and mRNA expression was elevated on the same days as caspase 3 protein and mRNA expressions^17^.

Scientific papers present some data concerning HIFs and TGF-β as therapeutic targets. The effect of TGF-β on parasitic diseases is investigated but the results are ambiguous^60^. Some papers show that TGF-β can reduce pathogen burdens as well as tissue injuries^61,62^, but there are also papers reporting that this cytokine can promote the survival and growth of parasites^63,64^. HIF1α inhibitors are also studied, albeit only for therapy of cancer, anemia, and vascular disease^58,59^. In future studies, it is worth to check the exact role of HIFs and TGF-β on the hosts infected with *Acanthamoeba* sp., because the modulation of these pathways can become therapeutic targets that will limit the degree of cell and organ damage.

Systemic acanthamoebiasis is an infection that concerns many organs, including the kidneys. The incidence of renal failure was confirmed through manifestations such as KIM-1, and MCP-1 in our previous study^16^. Based on our presented and previous studies, there is a link between *Acanthamoeba* sp. and increased oxidative stress, apoptosis^17^, hypoxia, angiogenesis, and collagen deposition in the kidneys of hosts. The research should be continued with (i) a larger study group, and (ii) some validation steps that confirm the consistency of different measurement techniques.

## Supplementary Information

Supplementary Information.

## Data Availability

Derived data supporting the findings of this study are available from the corresponding author (KK) on request.

## References

[1] Gao, H. et al. Role of hypoxia in cellular senescence. *Pharmacol Res.* **194**, 106841. 10.1016/j.phrs.2023.106841 (2023). 37385572 10.1016/j.phrs.2023.106841

[2] Rahane, D. et al. Hypoxia and its effect on the cellular system. *Cell Biochem Funct.* **42**, e3940. 10.1002/cbf.3940 (2024). 38379257 10.1002/cbf.3940

[3] Shu, S. et al. Hypoxia and hypoxia-inducible factors in kidney injury and repair. *Cells.* **8**, 207. 10.3390/cells8030207 (2019). 30823476 10.3390/cells8030207 PMC6468851

[4] Loboda, A., Jozkowicz, A. & Dulak, J. HIF-1 and HIF-2 transcription factors–similar but not identical. *Mol Cells.* **29**, 435–442. 10.1007/s10059-010-0067-2 (2010). 20396958 10.1007/s10059-010-0067-2

[5] Koh, M. Y. & Powis, G. Passing the baton: The HIF switch. *Trends Biochem. Sci.* **37**, 364–372. 10.1016/j.tibs.2012.06.004 (2012). 22818162 10.1016/j.tibs.2012.06.004 PMC3433036

[6] Kondo, K., Kim, W. Y., Lechpammer, M. & Kaelin, W. G. Jr. Inhibition of HIF2alpha is sufficient to suppress pVHL-defective tumor growth. *PLoS Biol.* **1**, E83. 10.1371/journal.pbio.0000083 (2003). 14691554 10.1371/journal.pbio.0000083 PMC300692

[7] Yuan, X., Ruan, W., Bobrow, B., Carmeliet, P. & Eltzschig, H. K. Targeting hypoxia-inducible factors: Therapeutic opportunities and challenges. *Nat Rev Drug Discov.* **23**, 175–200. 10.1038/s41573-023-00848-6 (2024). 38123660 10.1038/s41573-023-00848-6 PMC12337356

[8] Liu, J. et al. Hypoxia, HIF, and associated signaling networks in chronic kidney disease. *Int. J. Mol. Sci.* **18**, 950. 10.3390/ijms18050950 (2017). 28468297 10.3390/ijms18050950 PMC5454863

[9] Skuli, N. et al. Endothelial HIF-2α regulates murine pathological angiogenesis and revascularization processes. *J. Clin. Investig.* **122**, 1427–1443. 10.1172/JCI57322 (2012). 22426208 10.1172/JCI57322 PMC3314446

[10] Majmundar, A., Wong, W. & Simon, M. Hypoxia-inducible factors and the response to hypoxic stress. *Mol. Cell.* **40**, 294–309 (2010). 20965423 10.1016/j.molcel.2010.09.022 PMC3143508

[11] Liu, Z., Semenza, G. L. & Zhang, H. Hypoxia-inducible factor 1 and breast cancer metastasis. *J. Zhejiang Univ. B* **16**, 32–43 (2015). 10.1631/jzus.B1400221 PMC4288942 25559953

[12] Gilkes, D., Bajpai, S., Chaturvedi, P., Wirtz, D. & Semenza, G. Hypoxia-inducible factor 1 (HIF-1) promotes extracellular matrix remodeling under hypoxic conditions by inducing P4HA1, P4HA2, and PLOD2 expression in fibroblasts. *J. Biol. Chem.* **288**, 10819–10829 (2013). 23423382 10.1074/jbc.M112.442939 PMC3624462

[13] Mallikarjuna, P., Zhou, Y. & Landström, M. The synergistic cooperation between TGF-β and hypoxia in cancer and fibrosis. *Biomolecules* **12**, 635. 10.3390/biom12050635 (2022). 35625561 10.3390/biom12050635 PMC9138354

[14] Naas, S., Schiffer, M. & Schödel, J. Hypoxia and renal fibrosis. *Am J Physiol Cell Physiol.* **325**, C999–C1016. 10.1152/ajpcell.00201.2023 (2023). 37661918 10.1152/ajpcell.00201.2023

[15] Wang, Y. et al. Biological characteristics and pathogenicity of *Acanthamoeba*. *Front Microbiol.* **14**, 1147077. 10.3389/fmicb.2023.1147077 (2023). 37089530 10.3389/fmicb.2023.1147077 PMC10113681

[16] Kot, K. et al. Potential biomarkers in diagnosis of renal acanthamoebiasis. *Int J Mol Sci.* **22**, 6583. 10.3390/ijms22126583 (2021). 34205319 10.3390/ijms22126583 PMC8234237

[17] Kot, K. et al. The role of apoptosis and oxidative stress in the pathophysiology of *Acanthamoeba* spp. infection in the kidneys of hosts with different immunological status. *Parasit Vectors.* **16**, 445 (2023). 10.1186/s13071-023-06052-0. 10.1186/s13071-023-06052-0 PMC10693070 38041167

[18] Lin, Q. et al. Kidney injury molecule-1 expression in IgA nephropathy and its correlation with hypoxia and tubulointerstitial inflammation. *Am J Physiol Renal Physiol.* **306**, 885–895 (2014). 10.1152/ajprenal.00331.2013 24523388

[19] Eardley, K. S. et al. The relationship between albuminuria, MCP-1/CCL2, and interstitial macrophages in chronic kidney disease. *Kidney Int.* **69**, 1189–1197 (2006). 16609683 10.1038/sj.ki.5000212

[20] van Timmeren, M. et al. Tubular kidney injury molecule-1 in protein-overload nephropathy. *Am J Physiol Ren Physiol.* **291**, 456–464 (2006). 10.1152/ajprenal.00403.2005 16467126

[21] Kot, K. et al. Histological changes in the kidneys and heart in experimental Acanthamoebiasis in immunocompetent and immunosuppressed hosts. *Folia Biologica-Krakow.* **69**, 167–178 (2021).

[22] Robertson, H., Ali, S., McDonnell, B. J., Burt, A. D. & Kirby, J. A. Chronic renal allograft dysfunction: the role of T cell-mediated tubular epithelial to mesenchymal cell transition. *J Am Soc Nephrol.* **15**, 390–397. 10.1097/01.asn.0000108521.39082.e1 (2004). 14747385 10.1097/01.asn.0000108521.39082.e1

[23] Lanocha, N. et al. The occurrence *Acanthamoeba* [free-living amoeba] in environmental and respiratory samples in Poland. *Acta Protozool.* **48**, 271–279 (2009).

[24] Łanocha-Arendarczyk, N. et al. Expression and activity of COX-1 and COX-2 in *Acanthamoeba* sp.-infected lungs according to the host immunological status. *Int. J. Mol. Sci.* **19**, 121. 10.3390/ijms19010121 (2018). 29301283 10.3390/ijms19010121 PMC5796070

[25] DeMichele, E., Sosnowski, O., Buret, A. G. & Allain, T. Regulatory functions of hypoxia in host-parasite interactions: A focus on enteric, tissue, and blood protozoa. *Microorganisms.* **11**, 1598. 10.3390/microorganisms11061598 (2023). 37375100 10.3390/microorganisms11061598 PMC10303274

[26] Leitsch, D., Mbouaka, A. L., Köhsler, M., Müller, N. & Walochnik, J. An unusual thioredoxin system in the facultative parasite *Acanthamoeba castellanii*. *Cell Mol Life Sci.* **78**, 3673–3689. 10.1007/s00018-021-03786-x (2021). 33599799 10.1007/s00018-021-03786-x PMC8038987

[27] Grzenkowicz-Wydra, J. et al. Gene transfer of CuZn superoxide dismutase enhances the synthesis of vascular endothelial growth factor. *Mol Cell Biochem* **264**, 169181 (2004). 10.1023/b:mcbi.0000044386.45054.70 15544046

[28] Kumar, H. & Choi, D. K. Hypoxia inducible factor pathway and physiological adaptation: A cell survival pathway?. *Mediators Inflamm.* **2015**, 584758 (2015). 26491231 10.1155/2015/584758 PMC4600544

[29] Eckardt, K. U. et al. Role of hypoxia in the pathogenesis of renal disease. *Kidney Int.* **68**, S46–S51. 10.1111/j.1523-1755.2005.09909.x (2005). 10.1111/j.1523-1755.2005.09909.x 16336576

[30] Elias, R. M. et al. Oxidative stress and modification of renal vascular permeability are associated with acute kidney injury during *P. berghei* ANKA infection. *PLoS One.* **7**, e44004. 10.1371/journal.pone.0044004.E (2012). 22952850 10.1371/journal.pone.0044004 PMC3432099

[31] Park, M. K. et al. Induction of angiogenesis by malarial infection through hypoxia dependent manner. *Korean J Parasitol.* **57**, 117–125. 10.3347/kjp.2019.57.2.117 (2019). 31104403 10.3347/kjp.2019.57.2.117 PMC6526210

[32] Wiley, M. et al. *Toxoplasma gondii* activates hypoxia-inducible factor (HIF) by stabilizing the HIF-1alpha subunit via type I activin-like receptor kinase receptor signaling. *J Biol Chem.* **285**, 26852–26860. 10.1074/jbc.M110.147041 (2010). 20581113 10.1074/jbc.M110.147041 PMC2930684

[33] Arrais-Silva, W. W., Paffaro. V. A. Jr., Yamada, A. T. & Giorgio, S. Expression of hypoxia-inducible factor-1alpha in the cutaneous lesions of BALB/c mice infected with *Leishmania amazonensis*. *Exp Mol Pathol.* **78**, 49–54 (2005). 10.1016/j.yexmp.2004.09.002. 10.1016/j.yexmp.2004.09.002 15596060

[34] Kumar, V. et al. *Leishmania donovani* activates hypoxia inducible factor-1α and miR-210 for survival in macrophages by downregulation of NF-κB mediated pro-inflammatory immune response. *Front Microbiol.* **9**, 385. 10.3389/fmicb.2018.00385 (2018). 29568285 10.3389/fmicb.2018.00385 PMC5852103

[35] Werth, N. et al. Activation of hypoxia inducible factor 1 is a general phenomenon in infections with human pathogens. *PLoS One.* **5**, e11576. 10.1371/journal.pone.0011576 (2010). 20644645 10.1371/journal.pone.0011576 PMC2904385

[36] Alonso, D., Serrano, E., Bermejo, F. J. & Corral, R. S. HIF-1α-regulated MIF activation and Nox2-dependent ROS generation promote *Leishmania amazonensis* killing by macrophages under hypoxia. *Cell Immunol.* **335**, 15–21. 10.1016/j.cellimm.2018.10.007 (2019). 30384962 10.1016/j.cellimm.2018.10.007

[37] Degrossoli, A. et al. The influence of low oxygen on macrophage response to *Leishmania* infection. *Scand J Immunol.* **74**, 165–175. 10.1111/j.1365-3083.2011.02566.x (2011). 21517930 10.1111/j.1365-3083.2011.02566.x

[38] Lee, S. J. et al. Oxidized low-density lipoprotein stimulates macrophage 18F-FDG uptake via hypoxia-inducible factor-1α activation through Nox2-dependent reactive oxygen species generation. *J Nucl Med.* **55**, 1699–1705. 10.2967/jnumed.114.139428 (2014). 25214643 10.2967/jnumed.114.139428

[39] Guzy, R. D. et al. Mitochondrial complex III is required for hypoxia-induced ROS production and cellular oxygen sensing. *Cell Metab.* **1**, 401–408. 10.1016/j.cmet.2005.05.001 (2005). 16054089 10.1016/j.cmet.2005.05.001

[40] Chadha, A. & Chadee, K. The NF-κB pathway: Modulation by *Entamoeba histolytica* and other protozoan parasites. *Front Cell Infect Microbiol.* **11**, 748404. 10.3389/fcimb.2021.748404 (2021). 34595137 10.3389/fcimb.2021.748404 PMC8476871

[41] Bettadapura, M. et al. HIF-α activation impacts macrophage function during murine *Leishmania major* infection. *Pathogens.* **10**, 1584. 10.3390/pathogens10121584 (2021). 34959539 10.3390/pathogens10121584 PMC8706659

[42] Colegio, O. R. et al. Functional polarization of tumour-associated macrophages by tumour-derived lactic acid. *Nature.* **513**, 559–563. 10.1038/nature13490 (2014). 25043024 10.1038/nature13490 PMC4301845

[43] Koh, M. Y. & Powis, G. Passing the baton: The HIF switch. *Trends Biochem. Sci.* **37**, 364–372 (2012). 22818162 10.1016/j.tibs.2012.06.004 PMC3433036

[44] Younes, A. Angiogenesis in lymphoma: A short review. *Curr. Mol. Med.* **5**, 609–613 (2005). 16305487 10.2174/156652405774641098

[45] Epiphanio, S. et al. VEGF promotes malaria-associated acute lung injury in mice. *PLoS Pathog.* **6**, e1000916. 10.1371/journal.ppat.1000916 (2010). 20502682 10.1371/journal.ppat.1000916 PMC2873913

[46] Woodford, J. et al. Early endothelial activation precedes glycocalyx degradation and microvascular dysfunction in experimentally induced *Plasmodium falciparum* and *Plasmodium vivax* infection. *Infect. Immun.* **88**, 00895–00919. 10.1128/IAI.00895-19 (2020). 10.1128/IAI.00895-19 PMC7171246 32122938

[47] Verçosa, B. L. A. et al. MCP-1/IL-12 ratio expressions correlated with adventitial collagen depositions in renal vessels and IL-4/IFN-γ expression correlated with interstitial collagen depositions in the kidneys of dogs with canine leishmaniasis. *Mol Immunol.* **156**, 61–76. 10.1016/j.molimm.2023.02.010 (2023). 36889187 10.1016/j.molimm.2023.02.010

[48] Tanaka, T. & Nangaku, M. Angiogenesis and hypoxia in the kidney. *Nat Rev Nephrol.* **9**, 211–222. 10.1038/nrneph.2013.35 (2013). 23458926 10.1038/nrneph.2013.35

[49] Frangogiannis, N. Transforming growth factor-β in tissue fibrosis. *J Exp Med.* **217**, e20190103. 10.1084/jem.20190103 (2020). 32997468 10.1084/jem.20190103 PMC7062524

[50] de Caestecker, M. The transforming growth factor-β superfamily of receptors. *Cytokine Growth Factor Rev.* **15**, 1–11. 10.1016/j.cytogfr.2003.10.004 (2004). 14746809 10.1016/j.cytogfr.2003.10.004

[51] Isaka, Y. Targeting TGF-β signaling in kidney fibrosis. *Int. J. Mol. Sci.* **19**, 2532. 10.3390/ijms19092532 (2018). 30150520 10.3390/ijms19092532 PMC6165001

[52] Gu, Y. Y., Liu, X. S., Huang, X. R., Yu, X. Q. & Lan, H. Y. Diverse role of TGF-β in kidney disease. *Front. Cell Dev. Biol.* **8**, 123. 10.3389/fcell.2020.00123 (2020). 32258028 10.3389/fcell.2020.00123 PMC7093020

[53] Pereira, A. V. et al. Treatment with *Lycopodium clavatum* 200dH intensifies kidney and liver injury in mice infected with *Toxoplasma gondii*. *Arch. Immunol. Ther. Exp.* **68**, 1–14. 10.1007/s00005-020-00567-5 (2020). 10.1007/s00005-020-00567-5 31965304

[54] Kumar, V., Tiwari, N., Gedda, M. R., Haque, R. & Singh, R. K. *Leishmania donovani* infection activates toll-like receptor 2, 4 expressions and transforming growth factor-beta mediated apoptosis in renal tissues. *Braz. J. Infect. Dis.* **21**, 545–549. 10.1016/j.bjid.2017.04.007 (2017). 28606413 10.1016/j.bjid.2017.04.007 PMC9425502

[55] Alves, A. F., Pereira, R. A., de Andrade, H. M., Mosser, D. M. & Tafuri, W. L. Immunohistochemical study of renal fibropoiesis associated with dogs naturally and experimentally infected with two different strains of *Leishmania* (L.) *infantum*. *Int. J. Exp. Pathol.* **100**, 222–233. 10.1111/iep.12321 (2019). 31696580 10.1111/iep.12321 PMC6877998

[56] Cohen, E. P. et al. Detection and quantification of renal fibrosis by computerized tomography. *PLoS One.* **15**, e0228626. 10.1371/journal.pone.0228626 (2020). 32053617 10.1371/journal.pone.0228626 PMC7018060

[57] Hijmans, R. S. et al. Urinary collagen degradation products as early markers of progressive renal fibrosis. *J Transl Med.* **15**, 63. 10.1186/s12967-017-1163-2 (2017). 28320405 10.1186/s12967-017-1163-2 PMC5358042

[58] Bui, B. P., Nguyen, P. L., Lee, K. & Cho, J. Hypoxia-inducible factor-1: A novel therapeutic target for the management of cancer, drug resistance, and cancer-related pain. *Cancers (Basel).* **14**, 6054. 10.3390/cancers14246054 (2022). 36551540 10.3390/cancers14246054 PMC9775408

[59] Sharma, A., Sinha, S. & Shrivastava, N. Therapeutic targeting hypoxia-inducible factor (HIF-1) in cancer: Cutting Gordian knot of cancer cell metabolism. *Front Genet.* **13**, 849040. 10.3389/fgene.2022.849040 (2022). 35432450 10.3389/fgene.2022.849040 PMC9008776

[60] Deng, Z. et al. TGF-β signaling in health, disease, and therapeutics. *Signal Transduct Target Ther.* **9**, 61. 10.1038/s41392-024-01764-w (2024). 38514615 10.1038/s41392-024-01764-w PMC10958066

[61] Xu, X. et al. TGF-β1 improving abnormal pregnancy outcomes induced by *Toxoplasma gondii* infection: Regulating NKG2D/DAP10 and killer subset of decidual NK cells. *Cell Immunol.* **317**, 9–17. 10.1016/j.cellimm.2017.04.004 (2017). 28438315 10.1016/j.cellimm.2017.04.004

[62] Namangala, B., Sugimoto, C. & Inoue, N. Effects of exogenous transforming growth factor beta on *Trypanosoma congolense* infection in mice. *Infect. Immun.* **75**, 1878–1885. 10.1128/IAI.01452-06 (2007). 17261602 10.1128/IAI.01452-06 PMC1865695

[63] Barbosa, B. F. et al. IL10, TGF beta1, and IFN gamma modulate intracellular signaling pathways and cytokine production to control *Toxoplasma gondii* infection in BeWo trophoblast cells. *Biol. Reprod.* **92**, 82. 10.1095/biolreprod.114.124115 (2015). 25673564 10.1095/biolreprod.114.124115

[64] Walther, M. et al. Upregulation of TGF-beta, FOXP3, and CD4+CD25+ regulatory T cells correlates with more rapid parasite growth in human malaria infection. *Immunity* **23**, 287–296. 10.1016/j.immuni.2005.08.006 (2005). 16169501 10.1016/j.immuni.2005.08.006

