# Supplementary material for: Hypoxia and collagen deposition in the kidneys infected with Acanthamoeba sp
Source: Sci Rep. 2024 Nov 15;14:28096. doi: 10.1038/s41598-024-79848-4 (PMC11564555; doi:10.1038/s41598-024-79848-4)
Supplement: Supplementary file 1 — Supplementary Information. [file 41598_2024_79848_MOESM1_ESM.docx]

**Table S1.** Correlation analysis between the concentration of kidney injury molecule 1 (KIM-1) and the percentage of collagen fibers in the kidneys of mice infected with *Acanthamoeba* sp. A ‒ immunocompetent mice infected with *Acanthamoeba* sp., AS ‒ immunosuppressed mice infected with *Acanthamoeba* sp., C ‒ immunocompetent control mice, CS ‒ immunosuppressed control mice, dpi ‒ days post infection, R – rho, p – *p*-value, Spearman’s rank correlation coefficient analysis

|  | | | **KIM-1 concentration** | | | | | | | | | | | |
| --- | --- | --- | --- | --- | --- | --- | --- | --- | --- | --- | --- | --- | --- | --- |
|  |  |  | 8 dpi | | | | 16 dpi | | | | 24 dpi | | | |
|  |  |  | C | A | CS | AS | C | A | CS | AS | C | A | CS | AS |
| **The percentage of collagen fibers** | 8 dpi | C | R=-0.67  (p=0.22) |  |  |  |  |  |  |  |  |  |  |  |
|  |  | A |  | R=-0.70  (p=0.19) |  |  |  |  |  |  |  |  |  |  |
|  |  | CS |  |  | R=-0.56  (p=0.32) |  |  |  |  |  |  |  |  |  |
|  |  | AS |  |  |  | R=-0.41  (p=0.49) |  |  |  |  |  |  |  |  |
|  | 16 dpi | C |  |  |  |  | R=0.10  (p=0.87) |  |  |  |  |  |  |  |
|  |  | A |  |  |  |  |  | R=-0.16  (p=0.80) |  |  |  |  |  |  |
|  |  | CS |  |  |  |  |  |  | R=0.10  (p=0.87) |  |  |  |  |  |
|  |  | AS |  |  |  |  |  |  |  | R=-0.15  (p=0.80) |  |  |  |  |
|  | 24 dpi | C |  |  |  |  |  |  |  |  | R=0.70  (p=0.19) |  |  |  |
|  |  | A |  |  |  |  |  |  |  |  |  | R=-0.67  (p=0.22) |  |  |
|  |  | CS |  |  |  |  |  |  |  |  |  |  | R=0.82  (p=0.09) |  |
|  |  | AS |  |  |  |  |  |  |  |  |  |  |  | R=0.36  (p=0.55) |
